# Kevin Mitchell

**DOI:** 10.1192/bjb.2020.18

**Published:** 2020-04

**Authors:** Claire McKenna

**Figure:**
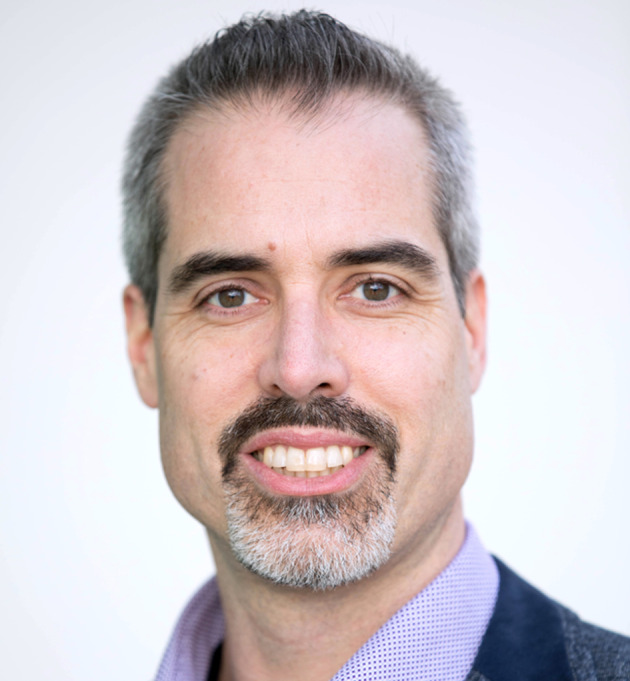

Professor Kevin Mitchell has a few pet peeves with the way neuroscience is represented in the media. To start, he highlights the siren call of fMRI scans that often adorn any remotely psychological article: ‘People demonstrably find that same article more convincing if it has a glowing brain in one corner of it than if it doesn't. So people are a bit susceptible, I think, to what we affectionately term “neuro-bollocks”’.

Mitchell is Associate Professor in Developmental Neurobiology and Genetics at Trinity College Dublin, where he is also Dean of Undergraduate Studies. His mission is to make complex neuroscience more accessible while resisting the temptations of neuro-reductionism. In his blog ‘Wiring the Brain’ (www.wiringthebrain.com), he writes spirited and forensic take-downs of overly hyped neuroscience research, as well as thoughtful philosophical explorations of the nature of the brain and mind. Blog titles include: ‘Is your future income written in your DNA?’ and ‘The murderous brain – can neuroimaging really distinguish murderers?’.

In 2018 he published *Innate*, an engaging popular science book exploring ‘how the wiring of our brains shapes who we are’, which has been praised by Stephen Pinker as a ‘new landmark’ in the old nature versus nurture debate.

Any other neuromyths he'd like to bust? He laughs, ‘There are so many!’. He is a critic of ‘blobology’ generally (the tendency to use blurry pictures of the brain to illustrate articles of little scientific value, such as ‘Your brain reacts to love like cocaine’), in part because it reflects a modular understanding of the way the brain works that bears no resemblance to reality.

One of his key messages is that nature is not obliged to make biology simple enough for us to understand. He does, though, think that public engagement by neuroscientists is vital, as ‘we see the effects of scientific illiteracy in lots of public policy’. He has several popular public talks on YouTube (including a TED talk titled ‘Who's in charge? You or your brain?’).

Mitchell fizzes with infectious enthusiasm for his subject. Two and a half hours later we're still talking and I feel I've just skimmed the surface. The themes of outdated traditions in neuroscience that try to localise brain functions (telling us nothing about the connectivity of the brain) and the lack of an underpinning philosophy of neuroscience are ones he continually comes back to. The way that neuroscience is taught explains the appeal of things like the decorative fMRI to professionals and public alike, he thinks. He emphasises that these scans are very indirect measures of neural activity on a background of constant endogenous activity. There is also the intuitive appeal of finding a ‘biomarker’ for mental illness.

## Biomarker research

Mitchell dismisses the idea that we have biomarkers for any neurodevelopmental or psychiatric conditions: ‘All that literature [on biomarkers] is polluted with false positives’. Small samples, lack of replication, statistical ‘fishing’ in an exploratory fashion are all major problems that undermine the validity of biomarker research. And, he says, ‘it gets worse if you add in the dimension of genomics to that, because now you have the enormous genomics space in which to search for covariates of neural activity or structure’.

So, no scans as diagnostic tests for mental illnesses then? He is bracingly sceptical:

‘There's not going to be a blood test. There's not going to be a brain scan. There's not going to be any other biomarker that captures those things, because they're looking at the wrong level. [Those conditions] are defined at the level of human behaviour […] Even if there's a dynamic neural state that underpins some aspect of psychosis that we both share, the way that that state looks in your brain may be very different from the way it looks in my brain because our brains are not the same. So I don't hold out much hope for, you know, getting to diagnostic biomarkers from that kind of imaging.’

## The neuro-hype of epigenetics

We turn next to epigenetics, a mechanism in molecular biology by which genes are ‘switched’ on and off and which is at risk of becoming ‘neuro-hype’. Epigenetics as a mode of intergenerational trauma transmission is very much a buzzword at present. Mitchell cautions against invoking sciency-sounding mechanisms to lend credibility to ‘nurture’ as a cause of psychological distress: ‘So people look at two fields – neuroplasticity/brain plasticity and now epigenetics – as some kind of a “get out of genetics free card”. I think people don't like the idea, some people anyway, that we are born with certain predispositions that are hard to change’.

Mitchell finds the concept of trauma transmission through epigenetic mechanisms implausible primarily because it suggests an overly simplistic relationship between genes and our psychological traits. Our experiences, he says, are expressed through changes in our neuroanatomy, not in our patterns of gene expression.

He sees in such flimsy claims a cautionary tale about the need for a different approach in science publishing: ‘There is a hype industry around science, which I think is corrosive. And I think scientists are willing participants in it in a way that I find more and more distasteful the older I get, because it does a massive disservice cumulatively to how science is understood by the general public because we have this constant hype’. He thinks his cynicism reflects a growing awareness in the research community of the need to focus on replicability and reproducibility and is hopeful things are changing for the better.

## ‘Biology is not just complicated physics’

Mitchell is most passionate when discussing the need to bring about a sea-change in the way neuroscience is taught and thought about. At this stage in his career he sees this as the biggest contribution he can make to his field.

By and large, he says, biological research is conducted in a theoretical vacuum, which means that a mechanistic understanding of how brains and minds work has become entrenched. Much neuroscience research is, he feels, linear and reductionist as a result:

‘We have one theory, which is the theory of evolution, which is great. And it does underpin everything. But it doesn't really explain how biological systems work and what they do […] There are lots of engineering principles and dynamic systems principles that we could be applying from those fields [from engineering and physics] that most biologists don't think about. It's just not the way that we approach things.’

He emphasises the need to get away from a modular understanding of how the brain works – the old ‘find the lesion’ trope – which works in neurology but not in psychiatry. ‘The connectivity across circuits is what's really important’, he says.

He thinks an understanding of neuroscience *is* important for psychiatrists but needs to be taught alongside complexity theory (understanding the dynamics of change in systems) so that we don't see brains as ‘passive stimulus–response machines’. He has a gift for the memorable tagline: ‘Biology is not just complicated physics!’, he quips.

## Undergraduates

Mitchell's genetics background influences his approach to his role as undergraduate dean. He says, ‘IQ scores, for example, are I think a measure not of potential but of achievement’. So two people with the same biological potential will perform differently depending on whether their environment allows them to thrive. He talks about the so-called ‘Matthew effect’ – the positive feedback loop between socioeconomic privilege, exam success and later career success that gets amplified across generations. Trinity College Dublin has pioneered novel approaches to making admissions criteria more equitable by accounting for social factors that affect a student's exam grades.

He is critical of commentators in the British press who use the partial heritability of intelligence to suggest that we live in a meritocracy and even to lend credence to eugenics: ‘It's a very Ayn Randian kind of idea that. Most of the people who've done well may have had some genetic capital in terms of talents and natural abilities but, of course, many of them also had lots of social capital and cultural capital’.

## Innate

In his book *Innate*, Mitchell argues strongly against the idea of the mind as a *tabula rasa* but he distinguishes his book from the work of people such as Robert Plomin, who also writes about the influence of DNA on our psychological traits. He agrees with Plomin that our traits are partly heritable but differs in how predictive he thinks that is.

He explains that the precise statistical meaning of ‘heritability’ is commonly misunderstood. It refers to the variance in a particular trait being due to genetic differences. This variance or ‘heritability’ is meaningful at a population level, but less useful when it comes to individuals. If, for example, intelligence is 50% heritable, it does not mean that 50% of your intelligence comes from your genes.

He is sceptical of the idea that genomic analysis or ‘polygenic risk scores’ in individuals can be used as a prediction of, for example, how intelligent that person will be, because of the massive spread in distribution of a particular trait across people with the same polygenic score.

Polygenic scores also have poor predictive value because of the nature of genetic variation. They capture a background of common mutations, each with a tiny effect on a trait, which account for about half of genetic effects, but ‘the rest will be from really rare newer mutations that have bigger effects but that kind of wink in and out of existence in a population because they get selected against’.

Another key theme in his work is that our DNA has distal and very indirect effects on the development of our brain. It is the multitudinous ‘noisy’ developmental processes between the transcription of the program encoded in our genome and the ‘wiring’ of our brain that are responsible for much of the variation in our traits. He says:

‘So one of the main points, I guess, of *Innate* that I was trying to make was that there's this source of variation in our psychological make-up that has gone largely unappreciated. It's not just genes and environment. There's this third source, third component of variation, this developmental variation that isn't due either to genetics or to environment. It's just the way that the development plays out during embryogenesis, during gestation and the way that it continues to play out over life.’

## Psychiatric genomics

All of this ‘noise’ in developmental variation is one of the reasons that Mitchell is guarded about the potential for genomics research to directly influence psychiatry. He points out that schizophrenia risk is only about 50% heritable but that doesn't necessarily mean that the other 50% of the variation is environmental: ‘A lot of the outcome may just be the random chance actually during development’.

Mitchell mistrusts much of the early work in psychiatric genetics, in particular the candidate gene association studies, in which researchers studied one or two genes at a time. He describes the statistical acrobatics in data analysis and publication bias towards positive results that threw up spurious associations between individual genes and psychological distress. Genome-wide association studies (GWAS) have shown candidate gene studies to be unreliable. The effects of genes implicated in the development of mental illness are mediated by the sum of interactions between probably thousands of different genes with different biochemical pathways.

So, has genetic research added to our understanding of the causes of mental illness? Mitchell thinks it has, but not in the way we'd hoped for. He cites the example that 80–90% of the variance in who develops autism is down to genetics, but most of the contributing variants are not inherited – so-called *de novo* variants.

Another key finding is that genetic risk factors overlap and are shared between multiple psychiatric and neurodevelopmental morbidities: ‘One of the things we've learned is that those sorts of [*de novo*] mutations can give rise in different people to autism or ADHD or epilepsy or intellectual disability or schizophrenia or bipolar disorder, depression or a whole range of things. So, they don't respect the diagnostic boundaries that we have’.

He feels that genetics research adds weight to the need to think about psychiatric disorders in dimensional terms but also in developmental terms: ‘You have two identical twins. As their brain is developing, what is the trajectory that leads one to develop schizophrenia and the other not? We can't just look at the genes and say these are genes for psychosis because that's not what the genes are doing’.

So far, so gloomy for a brave new world of psychiatric genomics leading to personalised medicine and new therapeutic targets. Genetics research will be useful for psychiatry in a probabilistic kind of way but he finds it difficult to see how it could be used to predict outcomes or therapies with certainty in individuals.

## Towards a unifying theory for neuroscience

Where then does the future lie for genetics research into psychiatric disorders? Mitchell stresses that genes are algorithms for making proteins. Human behaviour is not directly resultant from the activities of proteins; instead we perceive and think and feel with our neural circuits: ‘So, the way the neural circuits are organised is, for me, the proximal biological underpinning of the conditions. The genetic variations that led to those things being organised in that way are extremely distal causes […] So I think what we need to do is hand off to the neuroscientists’. Basically, he sees the biological causes of psychiatric disorders as a neuroscience problem: ‘I'm more optimistic about the idea of using genetic findings as a starting point to get at the neuroscience’.

He is particularly excited by the new field of optogenetics to help us study how discrete neural circuits work to influence animal behaviour in real time. He also hopes that the field of computational psychiatry will develop ‘so that we can develop a kind of a mature theory of what these circuits are doing in a complex dynamical systems kind of framework’.

He seems to bristle slightly at my suggestion that his approach to understanding the mind could be criticised as epiphenomenalism, and the associated problems with rooting thoughts, feelings and behaviours in biology. He appeals to holism: ‘I wouldn't use the word epiphenomena because that sounds a bit dismissive actually, but I would say emergent phenomena’. He emphasises that our cognitive and social development are crucial in understanding these phenomena: ‘We shouldn't think of nature and nurture as independent from each other, but highly interactive’.

He thinks neuroscience may be in the midst of a paradigm shift due to new technologies and mathematical tools that let us model the brain in a way that reflects its complexity.

So, I ask him, how long before the fruits of this new approach to neuroscience will benefit patients in psychiatry clinics? ‘Maybe if you ask me again in 5 years I'd be giving you, I think, a much more positive view of the importance of that. And if you ask me in 10 years, I'd be, I hope, pointing to areas where that understanding has led to some difference in the clinic’. He pauses. ‘Maybe 10 years is still too optimistic. Give me 20 years. Give us 20 years’. Not quite a headline that plays well to the gallery but a tonic in our era of spin.

